# New clinical insight in amyotrophic lateral sclerosis and innovative clinical development from the non-profit repurposing trial of the old drug guanabenz

**DOI:** 10.3389/fmed.2024.1407912

**Published:** 2024-06-10

**Authors:** Anna Ambrosini, Eleonora Dalla Bella, Maddalena Ravasi, Mario Melazzini, Giuseppe Lauria

**Affiliations:** ^1^Fondazione AriSLA ETS, Milan, Italy; ^2^Fondazione Telethon ETS, Milan, Italy; ^3^3rd Neurology Unit and ALS Centre, IRCCS ‘Carlo Besta’ Neurological Institute, Milan, Italy; ^4^IRCCS ‘Carlo Besta’ Neurological Institute, Milan, Italy; ^5^Department of Medical Biotechnology and Translational Medicine, University of Milan, Milan, Italy

**Keywords:** amyotrophic lateral sclerosis (ALS), drug repurposing, guanabenz, innovation, clinical development

## Abstract

Drug repurposing is considered a valid approach to accelerate therapeutic solutions for rare diseases. However, it is not as widely applied as it could be, due to several barriers that discourage both industry and academic institutions from pursuing this path. Herein we present the case of an academic multicentre study that considered the repurposing of the old drug guanabenz as a therapeutic strategy in amyotrophic lateral sclerosis. The difficulties encountered are discussed as an example of the barriers that academics involved in this type of study may face. Although further development of the drug for this target population was hampered for several reasons, the study was successful in many ways. Firstly, because the hypothesis tested was confirmed in a sub-population, leading to alternative innovative solutions that are now under clinical investigation. In addition, the study was informative and provided new insights into the disease, which are now giving new impetus to laboratory research. The message from this example is that even a repurposing study with an old product has the potential to generate innovation and interest from industry partners, provided it is based on a sound rationale, the study design is adequate to ensure meaningful results, and the investigators keep the full clinical development picture in mind.

## Introduction

Amyotrophic lateral sclerosis (ALS) is a severe neurological disease characterized by the degeneration of motor neurons. Although the ultimate cause of death derives from respiratory muscles failure, clinical manifestations are very heterogeneous, with anatomical distinction between bulbar versus spinal onset and speed of disease progression (1).

Biological studies suggested the pathophysiological involvement of various cellular pathways (2, 3). However, whether an impaired/dysfunctional mechanism is causative or a downstream effect that ultimately contributes to neuronal cell death remains challenging. Familial forms have been identified, implying a genetic origin of the disease in a small percentage (10%) of ALS cases, while the etiology of the disease is unknown in most patients and the interplay between dysfunctional pathways and exogenous risk factors may play a role. Close collaboration between basic and clinical researchers is encouraged to clarify the relationship between genetic and sporadic forms, correlate clinical heterogeneity with underlying disease mechanisms, develop informative preclinical models, and identify therapeutic targets.^1^

Given the large number of cellular pathways possibly involved in ALS, it is not surprising that several studies, both at preclinical and clinical level, have focused on known molecular targets to investigate the efficacy of new or existing drugs and to accelerate the development of therapies for ALS. However, despite much effort of academia and industry, most randomized clinical trials (RCTs) conducted over the last three decades have failed to demonstrate efficacy (4). Currently, only one drug - riluzole - has been approved by regulatory authorities worldwide, and few others are in advanced stages of development (3). The ALS scientific community has put a lot of effort into understanding the reasons for failure and improving the design of RCTs to ensure that any sign of efficacy of the putative drug is captured (2, 5, 6).

A white paper was recently published by an international group of experts including academics, industry, and patient representatives, to learn from experience and facilitate the translation of drug discovery into clinical development (7). The document proposed a framework of guiding principles, ranging from understanding the molecular basis of the disease, through drug discovery, to experimental medicine. Key successful factors include building a body of preclinical evidence in relevant models, tailored to the specific questions being asked. The advantages of phenotypic drug screening are also highlighted, with the possibility of drug repurposing and the potential for identifying novel targets, although it is acknowledged that this is labor intensive and target deconvolution can prove difficult. Finally, the lack of well-validated biomarkers to support drug discovery and development is identified as a relevant gap in ALS, hampering rapid assessment of target engagement and efficacy in preclinical and clinical studies (7).

Following the guiding principles of this white paper, we have reviewed here an Italian case study based on drug repurposing with guanabenz, an old FDA-approved alpha-2 adrenergic receptor agonist, that was tested in a phase 2 RCT conducted in patients with ALS (8). This was an academic pilot study mainly supported by Fondazione italiana di ricerca per la Sclerosi Laterale Amiotrofica (AriSLA), the Italian funding agency dedicated to research on ALS (9),^2^ as the result of one of its competitive calls for research projects. The trial design was based on 4 arms, testing 3 different doses of guanabenz against placebo (10), with efficacy and safety as endpoints. Although the study confirmed the hypothesis of non-futility for the 2 highest doses of treatment in patients with bulbar onset (8), further clinical development could not be pursued for several reasons herein briefly discussed. However, the study generated relevant information that is now contributing to innovative therapeutic developments and to advance knowledge of the disease. This example highlights the contribution of an old drug repurposing trial to innovation, and it challenges the concept of ‘successful trial’, regardless the possibility to make the treatment available to the patient population of interest.

## Results

### From preclinical target validation to a phase 2 clinical trial

Endoplasmic reticulum (ER) stress and the unfolded protein response (UPR) are part of a physiological protective mechanism to regulate cellular proteostasis by upregulating specific genes and inhibiting protein translation. Under transient stress events, the UPR allows the ER to return to physiological conditions. Under prolonged stress conditions, additional pathways are triggered, involving phosphorylation of the eukaryotic translation initiation factor 2A (eIF2α) (11), which causes a reduction in the flux of proteins into the ER followed by activation of a negative feedback loop by the phosphatase complex protein phosphatase 1 regulatory subunit 15A (PPP1R15A), which restores proteostasis. PPP1R15A’s key role was suggested by studies showing that its genetic inactivation led to a significant increase of SOD1^*G*93*A*^ and SOD1^*G*85*R*^ mice survival (12, 13). If stress conditions are not resolved, the UPR leads to apoptosis and cell degeneration. The latter has been described in ALS (14, 15) as the outcome of prolonged ER stress due to the accumulation of misfolded proteins such as, for instance, transactive response DNA-binding protein 43 (TDP43) (16), fused in sarcoma (FUS) (17), or superoxide dismutase type 1 (SOD1) (18). Persistent ER stress has been shown to contribute to neurodegeneration both in animal models and in cells derived from patients with sporadic (18) or familial (19) ALS. In addition, aggregates derived from misfolded ALS-related proteins trigger a ‘prion-like’ diffusion between cells, a mechanism that occurs at the level of the ribosomal RNA V-subunit folding, further contributing to impaired protein production and ultimately neuron death (20, 21).

Guanabenz, an old alpha-2-adrenergic receptor agonist used as an antihypertensive drug, has been shown to modulate the transcription factor-dependent synthesis of PPP1R15A and to reduce the activity of the eIF2α phosphatase complex, resulting in increased levels of phosphorylated eIF2α and reduced ER overload both *in vitro* cell cultures (22) and *in vivo* studies in *C. elegans* and Dario rerio ALS models (23). Importantly, the constitutive form of the eIF2α phosphatase PPP1R15B was not affected, avoiding persistent eIF2α phosphorylation and complete impairment of protein translation (22). Guanabenz also modulated ribosomal folding, reducing prion-like propagation in yeast, Drosophila and mouse models (24). These effects were also demonstrated in various ALS mouse models, where the molecule ameliorated the disease progression (25–27). The modulation of protein misfolding and the adrenergic activity of the drug were based on independent mechanisms of action (28). Overall, *in vitro* and *in vivo* evidence suggested that the UPR pathway may be a potential therapeutic target for ALS and provided a robust rationale for considering repurposing of guanabenz in ALS.

### The ‘ProMISe’ trial

A multicentre, randomized, double-blind phase 2 trial with a futility design was developed to test efficacy and safety of guanabenz in ALS patients (‘ProMISe’ trial) (10). The study design was based on 4 arms (1:1:1:1 ratio), with 3 different doses of guanabenz: 16, 32, 64 mg taken twice daily plus 100 mg riluzole daily versus 100 mg riluzole daily alone (placebo). The placebo arm was meant only to compare safety and tolerability. The null hypothesis of the study was that guanabenz would reduce the proportion of patients who progressed to a higher disease stage at 6 months by at least 35% compared to their baseline and a historical cohort of 200 Italian patients. The functional measures included, among others, the ALS-Milano-Torino Staging (MITOS) system, which was considered suitable to verify the null hypothesis.

Eligible participants, aged ≥18 years, had to be diagnosed with a sporadic or familial form of ALS with onset <18 months before enrolment. A total of 201 patients meeting the inclusion criteria were randomized. Their demographics, disease characteristics, and progression rate were not significantly different from the historical cohort. Details of the study can be found in (8).

The study was coordinated by the IRCCS Foundation ‘Carlo Besta’ Neurological Institute, Milan, Italy, and involved 22 trial sites across Italy (8) to achieve the patient number required by the sample size calculation. This implied several management issues: (i) approval by all ethics committees took a long time, prolonging the randomisation period and the overall duration of the study; (ii) it required training among centres to ensure harmonization of outcome measures and procedures; (iii) it required a strong coordination and monitoring activities. The need to involve multiple trial centres to ensure statistical validation is a common condition in rare diseases and is not a unique feature to drug repurposing trials. However, as an academic study funded by a small charity, it was found that there was insufficient funding to cover all management needs for such a large multicentre trial and partner sites were not adequately supported.

But the biggest hurdles came from the drug itself. While the trial was in preparation, guanabenz had been withdrawn from the market. The principal investigator managed to find a company to manufacture - and donate - the active product, the investigational medicinal product, and placebo, knowing from the outset that, even if the outcome was positive, the drug would not be available for further clinical development. During the trial, the alpha-2 adrenergic antihypertensive effect of the drug proved to be a major limitation, accounting for higher dropout rates among individuals in the two higher dose arms. Although this was a confounding factor in the final analysis, the trial hypothesis of non-futility was reached for the two higher dose groups, with a significantly lower proportion of patients who progressed to a higher stage of disease than in the lowest dose or the placebo group, and the historical controls. This difference was due to the striking response of patients with bulbar onset (none of them in these two groups progressed to a higher stage of disease during the 6 months of treatment, compared with 50% in the lowest dose group, 36% in the placebo group, and 43% in the historical cohort), suggesting important differences between patients with bulbar and spinal onset after modulation of this molecular target, given that no differences were observed between groups of patients with spinal onset (8). It is possible that without the confounding factor of poor tolerability of the drug the difference between the spinal and bulbar onset groups might have been even more pronounced.

### Innovative outcomes

Although further clinical development with guanabenz was not an option, due to unavailability of the drug and, more importantly, the poor tolerability of the effective doses in a patient population that does not suffer from hypertension, the ProMISe trial provided relevant information that opened new innovative therapeutic scenarios for ALS.

The primary hypothesis verified with the pioneering non-futility statistical method was achieved for patients with bulbar onset in the two higher dose groups and supported the rationale for the UPR pathway as a potential therapeutic target in ALS. This led to the identification of sephin1, a synthetic derivative of guanabenz without the alpha-2 adrenergic component (27, 29) as a potential replacement for guanabenz. This molecule, renamed icerguastat, had already underwent a phase 1 trial in healthy volunteers^3^ (30) and, after the outcomes of the ProMISe study, a multicentre and multinational phase 2 trial was started, which is currently underway in Italy and France with the ‘Carlo Besta’ Institute in Milan coordinating the Italian trial sites (TRIALS protocol)^4^ (31).

Icerguastat is a close derivative of guanabenz, differing only by one chlorine atom. These two small molecules share the same pharmacological targets and metabolic profile, but icerguastat lacks hypotensive activity, thus overcoming guanabenz’s limitation. Icerguastat has been granted as Orphan Drug Designation for ALS treatment by FDA and EMA [DRU-2021-8683;^5^ (32), and EMA/OD/0000079683^6^, respectively, (33)] and is administered by oral route. By inhibiting PPP1R15A phosphatase complex activity, it ensures re-initiation of translation (34) and prolongs eIF2 phosphorylation, leaving cells more time to cope with the stress (27). This could be a relevant neuroprotective effect as in post-mortem tissues of ALS patients integrated stress response markers were found together with altered ER cisternae and secretory pathway (35) and increased expression of PERK, eIF2α, ATF4 and CHOP (15, 36–38).

Based on the lessons learned from the guanabenz trial, the new study included only ALS patients with bulbar onset, comparing a drug regimen of 25 mg twice a day + 100 mg riluzole versus riluzole alone (2:1 ratio; approximately the lowest effective dose of the guanabenz trial). The primary endpoint of the ongoing trial is safety, while the secondary endpoint evaluates efficacy using, among other measures, the progression to a higher stage of disease (MITOS system) as previously assessed in the ProMISe trial.

The ProMISe study also analyzed potential differences in biomarker levels between arms, without finding significant changes in the neurofilament component (8). However, the evaluation of miRNA transcriptional levels, adopted as biological evidence of target engagement, highlighted differences between ALS patients with bulbar versus spinal onset. This has led to a new research project, selected for funding by AriSLA (39) 2023 competitive call, which will investigate miRNA differences in biological samples, including those collected as part of the ProMISe study. Sophisticated molecular analyses will be used to compare the two groups of patients with each other and with samples from healthy individuals. In addition, machine learning analyses will be used to compare molecular and clinical profiles, challenging the current paradigms of ALS classification.

This case study is illustrated in Table 1 according to the framework used in the 2023 ALS white paper (7).

**TABLE 1:** Key features of the development roadmap for guanabenz in ALS from preclinical to clinical studies.

Case study: guanabenz repurposing trial in ALS:
**Rationale:**
ER stress and the UPR are persistent conditions in ALS, due to the accumulation of misfolded proteins such as TDP43, FUS, or SOD1. This mechanism involves phosphorylation of the translation factor eIF2α that reduces the flux of proteins into the ER, then followed by a negative feedback loop involving the PPP1R15A phosphatase complex, which restores proteostasis. ALS aggregates also trigger a ‘prion-like’ diffusion at the ER level, further contributing to neurodegeneration. Guanabenz reduced ER overload by modulating the synthesis of PPP1R15A, thereby increasing levels of phosphorylated eIF2α. Guanabenz also reduced the ‘prion-like’ diffusion of aggregated proteins.
**Preclinical studies:**
• ER stress and UPR have been shown to contribute to neurodegeneration in animal models and in cells derived from patients with sporadic and familial ALS. • Guanabenz reduced the ER overload both *in vitro* cell cultures and *in vivo* studies in C. elegans and Dario rerio ALS models. • Guanabenz modulated ribosomal folding, reducing ‘prion-like’ propagation in yeast, Drosophila and mouse models. These effects were also demonstrated in various ALS mouse models, where the molecule ameliorated the disease progression.
**Clinical studies: the ‘ProMISe’ trial:**
A multicentre, randomized, double-blind phase 2 trial with a futility design was developed to test the efficacy and safety of guanabenz in patients with ALS, with 3 different doses of the drug and placebo arms. • The primary hypothesis of non-futility was achieved in the two higher dose groups in ALS patients with bulbar onset supporting the rationale for the UPR pathway as a potential therapeutic target in ALS. • The alpha-2 adrenergic component of guanabenz reduced the tolerability of guanabenz in ALS patients. • New knowledge of the disease was generated by the trial, particularly highlighting differences between patients with the bulbar versus spinal onset.
**Implication for future studies:**
• A new multicentre and multinational phase 2 RCT with icerguastat, a synthetic derivative of guanabenz without the alpha-2 adrenergic component, is underway with a design based on information from the ProMISe study. The new study included only ALS patients with bulbar onset. • New research studies are ongoing to characterize miRNA biomarkers in ALS. Sophisticated molecular analyses will be used to compare ALS patients with bulbar versus spinal onset. Innovative machine learning analyses will be used to compare molecular and clinical profiles, challenging the current paradigms of ALS classification.

ALS, amyotrophic lateral sclerosis; ER, endoplasmic reticulum; UPR, unfolded protein response; TDP43, transactive response DNA-binding protein 43; FUS, fused in sarcoma; SOD1, superoxide dismutase type 1; eIF2α, eukaryotic translation initiation factor 2A; PPP1R15A, protein phosphatase 1 regulatory subunit 15A; RCT, randomized clinical trial.

## Discussion

A large body of literature highlights the strategic importance of drug repurposing in rare diseases. Major advantages include the availability of a wealth of information on the drug’s manufacturing processes, and clinical knowledge on its safety profile, pharmacokinetics, and effective doses. However, it is relatively rare for repurposed medicines to be approved at the request of industry. This may be due to expiring intellectual property rights or lack of incentives that make the burden of going through the necessary development unattractive to industry, particularly if the target is a rare or ultra-rare disease. As a result, clinical development using this approach is more frequently attempted by academics, often together with non-profit organizations (40, 41), seeking an opportunity to test a therapeutic approach for an orphan disease, also taking advantage of similarities with other disorders (42). Although it is easier to do this with a drug already on the market, there are still many barriers to clinical development.

We have presented here the example of the ProMISe trial, an academic study funded by AriSLA, a non-profit foundation with a specific interest in ALS, to illustrate some of the obstacles encountered and to comment on the key success factors that contributed to the clinical progress (Table 1). Among the obstacles, the limited financial resources available to carry out a large multicentre study have been a critical issue. Careful coordination and strong motivation on the part of all participating sites enabled to overcome this problem, but this should not be underestimated when planning a non-profit trial, to ensure the optimal study conduct and quality of data collected.

In line with the guiding principles for clinical development discussed in a recent ALS white paper (7), some key aspects that contributed to the success and informative value of the ProMISe trial have been identified: i) the rationale for the selection of the molecular target and the active molecule was sufficiently robust and independently validated by different groups in multiple cellular and animal models; ii) the study design was based on the pioneering futility model and tailored to the hypothesis to be demonstrated; iii) selected outcome measures were appropriate; iv) the pilot trial was considered as part of a clinical development process.

On the latter point, a major obstacle is, in general, represented by the fact that academics do not have the expertise, nor the interest or the legal status, to become marketing authorisation holder themselves. Very often, researchers focus on the clinical validation of an identified molecular target to provide a proof of concept of efficacy, more out of curiosity than awareness of the long process required to bring the drug to market. Even in the best-case scenario, at some point in development they will still need to engage an industry partner to proceed further with a pivotal trial and marketing registration.

The demonstration of guanabenz’s effect in bulbar onset ALS prompted an innovative RCT design involving only patients presenting with that specific phenotype. Within the huge heterogeneity of ALS, whose multiple phenotypes have different course leading to death in a temporal range from 1 to more than 10 years after onset (43), bulbar onset is the most homogeneous phenotype both in terms of progression (44) and neuropathological features (45). It accounts for about 30% of all patients and is diagnosed by validated diagnostic criteria (43, 46). Albeit more frequent in patients with C9orf72 hexanucleotide repeat expansion (47), bulbar onset is not correlated to a specific genotype, occurring with similar frequency both in patients carrying gene mutations and in negative cases (43, 48, 49).

In the last 30 years, the concept of ALS as a unique disease (43, 46, 49, 50) drove the design of RCTs, most of which have failed (4). This view implies that any patient, regardless of the phenotype at onset and its course, could share the same pathophysiology and have the same potential to respond to a disease-modifying drug. The ProMISe study suggested that it may not be the case, as anticipated by the trial on riluzole showing a median survival improvement by 2.8-fold in bulbar onset patients without any advantages in spinal onset patients (51).

Innovation and generation of new knowledge are important factors in facilitating the involvement of an industry partner. The ProMISe study showed that it was not possible to repurpose guanabenz for ALS due to its limited tolerability and its withdrawal from the market. However, the information gained helped engage a company in a new development pathway with a derivative molecule without the adverse effects of guanabenz and provided important guidance for the design of a new trial that focused on bulbar onset patients. Whether the new compound’s greater target specificity is sufficient to replicate the positive effects seen with guanabenz requires confirmation from the new study.

Since 2018, AriSLA has not admitted interventional studies in its annual calls, as the costs of these studies are not sustainable for the Foundation, while its calls are open to basic/preclinical research and observational clinical studies ([9]; see text footnote 3). Inspired by the ALS strategic plan published by the National Institutes of Health U.S. National Institute of Neurological Disorders and Stroke (52),^7^ in 2023 AriSLA launched its new strategic plan, setting new priorities in its calls for studies aimed at understanding ALS heterogeneity in humans as well as in preclinical models. The new research study on biomarkers including samples collected from the guanabenz trial responded to the priorities of the call and scientific quality requirements and was selected by the AriSLA’s peer review panel as part of the 2023 selection process.

## Conclusion

The ProMISe trial was successful not only because it confirmed the null hypothesis, but also because of the knowledge it generated about the disease. Even though the repurposing of guanabenz could not be pursued, the study opened innovative avenues that are now under clinical development in ALS. This should be seen as an important message to encourage academics to seize every opportunity to explore drug repurposing in rare diseases, provided the preclinical rationale is sufficiently robust, the study design is tailored to adequately address the underlying hypothesis and be informative, and there is a vision of the study as part of a long-term clinical development process.

## Author contributions

AA: Conceptualization, Writing – original draft, Writing – review and editing. EDB: Investigation, Validation, Writing – review and editing. MR: Project administration, Writing – review and editing. MM: Resources, Supervision, Writing – review and editing. GL: Conceptualization, Investigation, Validation, Writing – review and editing.

## Footnotes

1
https://www.ninds.nih.gov/about-ninds/strategic-plans-evaluations/strategic-plans/amyotrophic-lateral-sclerosis-als

2
https://www.arisla.org/en/

3
https://clinicaltrials.gov/study/NCT03610334?term=ifb-088&rank=2

4
https://clinicaltrials.gov/study/NCT05508074?cond=Amyotrophic%20Lateral%20Sclerosis&term=ifb-088&rank=1

5
https://www.accessdata.fda.gov/scripts/opdlisting/oopd/detailedIndex.cfm?cfgridkey=868321

6
https://www.ema.europa.eu/en/medicines/human/orphan-designations/eu-3-22-2618

7
https://www.ninds.nih.gov/sites/default/files/documents/ALS%20Strategic%20Plan_01_19_23_508C.pdf
